# Investigating barriers and challenges to the integrated management of neglected tropical skin diseases in an endemic setting in Nigeria

**DOI:** 10.1371/journal.pntd.0008248

**Published:** 2020-04-30

**Authors:** Kingsley N. Ukwaja, Chukwuka Alphonsus, Chinwe C. Eze, Linda Lehman, Ngozi Ekeke, Charles C. Nwafor, Joy Ikebudu, Moses C. Anyim, Joseph N. Chukwu

**Affiliations:** 1 Department of Internal Medicine, Alex Ekwueme Federal University Teaching Hospital, Abakaliki, Ebonyi State, Nigeria; 2 Medical Department, German Leprosy and TB Relief Association, Enugu State, Nigeria; 3 American Leprosy Missions, Greenville, South Carolina, United States of America; Liverpool School of Tropical Medicine, UNITED KINGDOM

## Abstract

**Background:**

There is a dearth of experience in and evidence for cost-effective integrated community-based management of skin neglected tropical diseases (NTDs). The objective of this study was to assess the knowledge, attitude and care-seeking practices including self-care with a view to introducing appropriate community-based interventions for skin NTDs in an endemic setting in Southern Nigeria.

**Methods/Principal findings:**

This exploratory study adopted a mixed-methods design consisting of cross-sectional surveys of community members and health workers using interviewer-administered questionnaires; and focus group discussions (FGDs) with community members, health care workers and patients with NTDs in Anambra State, Nigeria. The survey was completed by 353 community members (61.8% female) and 15 health care workers (100.0% female). A total of 52 individuals participated in six FGDs. Of the community members, 236 (66.9%) had heard or seen a case of leprosy; 324 (91.8%) and 131 (37.5%) had heard or seen a case of Buruli ulcer and lymphatic filariasis, respectively. Again, 213 (60.3%) of the respondents reported that the diseases were caused by witchcraft or curse. As regards prevention, 241 (68.3%) suggested avoiding handshake with affected persons. Up to 223 (63.2%) of respondents strongly agreed to the seriousness of skin NTDs in their community. Meanwhile, 272 (77.1%) of the respondents believed that the transmission of these skin NTDs can be prevented. Furthermore, 324 (91.7%) desired active community engagement for control of skin NTDs. Regarding community care seeking practices, 197 (55.8%) would first visit the health centre/hospital, followed by 91 (25.8%) traditional healer/herbalist and 35 (9.9%) pharmacy/patent medicine vendor if they develop a skin NTD. Overall, 332 (94.1%) of respondents expressed interest in being taught self-care practices for skin NTDs. Out of 15 healthcare workers, 13 (86.7%) were able to correctly diagnose two of these skin NTDs and 10 (66.7%) would encourage patients to practice self-care. Prominent themes in the FGDs were belief in witchcraft and herbal remedies; as well as the occurrence of physical, social and economic distress.

**Conclusions:**

Our study helped quantify the information gaps that need to be addressed in order to create demand for integrated skin NTDs services in an endemic setting in Nigeria. Individual, structural and socioeconomic challenges to access and delivery of services were identified. Community and health care workers’ empowerment and engagement through outreach and regular training, respectively may alleviate these challenges.

## Author summary

Community perceptions of causation of neglected tropical diseases (NTDs) of the skin may play an important role in access to or utilization of health services. The World Health Organization (WHO) has recommended empowerment of populations affected by or at risk of NTDs in control interventions. Furthermore, the WHO recommends that social mobilisation needs to be maintained in order to create demand for integrated management of skin NTDs and to address specific community aspects and concerns related to the diseases. There are no studies on community knowledge, attitudes and practices (KAP) on skin NTDs co-occurring in the same community in Nigeria. We surveyed community members and health workers and also held group discussions with community members, health workers and individuals with lymphatic filariasis and Buruli ulcer in order to assess their understanding of the causes, treatment and effects of the skin NTDs (leprosy, Buruli ulcer and lymphatic filariasis) which were all occurring in the study communities. There was a shared understanding that these NTDs were caused by germ/infection or through witchcraft/curse/poison. Also, a substantial proportion of the community believed that these conditions are not amenable to treatment. The focus group discussions reinforced these findings.

## Introduction

Neglected tropical diseases (NTDs) are a diverse group of diseases and conditions prevailing in tropical and subtropical regions which affect over one billion people [1–2]. NTDs disproportionately affect populations living in poverty, having poor sanitation and living in close contact with infectious vectors and in proximity with domestic animals. Furthermore, NTDs cause approximately 534 000 deaths annually–accounting for about 10% of deaths caused by the global burden of infectious and parasitic diseases [2–3]. Also, they are associated with high level of deformity and disability, contributing to 25% of the global Disability Adjusted Life Years (DALYs) [3–4]. In addition, the long-standing nature of many NTDs perpetuates the cycle of poverty and imposes a heavy burden on already weak and over-stretched health systems [2]. The importance of NTDs as diseases of poverty has led to their consistent acknowledgement over the last three decades in the global public health agenda [5]. Currently, the control of NTDs is included in the Sustainable Development Goals–item 3 of goal 3 stipulates ending the epidemics of NTDs by 2030 [6].

The World Health Organization (WHO) African Region accounts for almost half of the global burden of NTDs [7]. Indeed, dracunculiasis, Buruli ulcer and human African trypanosomiasis affect only or mainly the African continent [7]. Furthermore, all 47 countries in the African Region are endemic for at least one NTD and 37 of them (79%) are co-endemic for at least five of these diseases [7–9]. In terms of control measures, the 20 diseases and conditions that constitute the NTDs are grouped into two: those that are amenable to preventive chemotherapy (PC) i.e., PC-NTDs such as lymphatic filariasis (LF), onchocerciasis, schistosomiasis, etc; and those that are addressed through case management (CM), i.e., CM-NTDs such as leprosy, human African trypanosomiasis and Buruli ulcer (BU) [7–9]. Thus, effective control of NTDs can be achieved with the use of large-scale delivery of preventive chemotherapy (PC) or intensified disease management (IDM) or both, as is the case for some diseases such as LF, trachoma, and yaws [7–9].

Among all of the African nations, Nigeria has the greatest number of people infected with NTDs–accounting for one-fourth or more of the global NTD disease burden and 13 of the 20 NTDs are endemic in the country [10]. While several efforts and progress have been made in the integrated delivery of preventive chemotherapy for PC-NTDs, not much progress has been made in integrating intensified disease management for the CM-NTDs despite the 66th World Health Assembly call for Member States to intensify and integrate measures against NTDs [5,10]. The WHO's Department of Control of NTDs (WHO/NTD) recently rolled-out plans to promote an integrated strategy for the skin NTDs requiring IDM [11]. This is because in addition to high burden of disability and debilitating deformities, skin NTDs also result in high levels of stigmatization, discrimination, and psychological distress, which contribute to suffering and may affect health-seeking behaviours and adherence to treatment [8, 12–13]. Furthermore, most management for CM-NTDs has been hospital based and require much cost and long hospital stay which causes high burden on many patients [11]. However, studies have shown that community-based care where patients are taught self-care is cost-effective and promotes adherence with better outcome [11]. Integrating management of skin NTDs will require adequately trained individuals to suspect and clinically diagnose the diseases [14]. Furthermore, health workers need to be equipped to adequately manage as well as train skin NTD patients and their carers on self-care practices [14].

Community perceptions of causation of NTDs may play an important role in access to or utilization of health services [15–16]. One of the key guiding principles of the WHO African Region Strategic Plan for NTDs is empowerment of people and communities through the involvement of populations affected by or at risk of NTDs in control interventions [7]. Furthermore, the WHO recommends that social mobilisation needs to be maintained in order to create demand for integrated management of skin NTDs and to address specific community aspects and concerns related to the diseases [11]. There are no studies on community knowledge, attitudes and practices (KAP) on the co-occurrence of skin NTDs (i.e., leprosy, BU and LF) in Nigeria. However, this evidence is crucial in designing cost-effective community engagement strategies for integrated IDM for these skin NTDs and to support elimination efforts for the skin-NTDs. In 2017, a project funded by American Leprosy Missions (ALM) was set up by the German Leprosy & TB Relief Association with the aim of implementing an integrated morbidity management and disability prevention for the skin-NTDs (leprosy, BU and LF) in an endemic setting of Nigeria. Before project implementation began, a survey was carried-out which aimed to collect both quantitative and qualitative data on local knowledge, attitudes and care-seeking practices including self-care concerning skin NTDs in the setting.

## Materials and methods

### Study setting

The study was carried-out in Okpoko and Ogbakuba communities in Ogbaru Local Government Area (LGA) of Anambra State, South-East Nigeria. Ogabru is one of the 21 LGAs (equivalent to districts) of the State, with a total population of 294,342. The LGA is endemic for NTDs and is located in the tropical rain forest belt–along the south-eastern bank of the River Niger. The LGA is known to be co-endemic for LF, BU, leprosy, schistosomiasis and soil-transmitted helminthiasis The NTDs programmes in the LGA are co-ordinated by a Local Government NTDs coordinator.

The two communities were chosen due to the presence of health workers and community volunteers with previous experiences in the management of BU and leprosy. Four primary health care centres (PHCs) in the communities served as the main project site [17]. There are no reliable data on the burden of BU, leprosy and LF in the study communities. About two years before our survey we carried out a case finding search for BU in the communities and found over 30 cases [17]. Also, data available in Anambra State TB, Leprosy and BU Control Programme indicate that over 20 leprosy cases has been notified from the survey communities in the three years preceding the study. Following the treatment (with medications) of patients with these NTDs, health workers in the study health centres complained that some of the patients returned with impairments/disability; and they also had reported having a few patients with LF who had impairments and disability.

Nevertheless, while we have held trainings with health workers in the State (Anambra State, where the study communities are located) about the identification and treatment of BU, we are not aware of, and this survey did not specifically assess whether the health workers have received any recent/formal training on the management of impairments associated with LF, leprosy and BU after the treatment of these diseases. These skin NTDs have the potential to cause chronic ill-health and long-term disability even after successful treatment of the underlying cause. E.g., a patient with treated lepromatous leprosy may have disabling limb deformity and oedema. A patient with treated LF may suffer with debilitating lymphoedema, and a patient with treated BU may present with deformity or contractures. We have not offered and there was no specific training for the management of these impairments for health workers in the study setting prior to the survey.

These observations informed the design of a project on integrated morbidity management and disability prevention for individuals with BU, leprosy and LF; and this survey was carried out to gain a better understanding of the situation in the study communities.

### Study design

This exploratory study adopted a mixed-methods design using quantitative and qualitative methods. The main methods used were descriptive cross-sectional surveys of community members and health workers using semi-structured interviewer-administered questionnaires, and focus group discussions (FGDs) sessions with community members, health care workers and patients with an NTD. The study was carried out between November and December 2017, and sought to assess understanding of the three-common skin NTDs (leprosy, BU and LF) in the communities.

### Study population and sampling

In the community survey, the primary sampling units were the 263 settlements also called census enumeration areas (EAs) in the study communities. There are 12 EAs in Ogbakuba and 251 in Okpoko. A total of 23 EAs were selected for the survey; 4 in Ogbakuba and 19 in Okpoko were selected through systematic sampling. The number of individuals interviewed in the two communities varied based on their population size projected from the 2006 census. In the chosen EAs, systematic random sampling was used to select 10–25 households each until the required sample size was reached. In each selected house, either the head of the household or the available most senior adult male or female resident was interviewed at home. In the health workers survey, all seven health workers in the project health facilities were selected for the survey and another eight were interviewed from two non-project PHCs from two other communities.

The FGD was based on the Explanatory Model Interview Catalogue (EMIC) framework for cultural epidemiology [18], and prior ethnographic studies [3] on some skin NTDs. The instruments were developed in English, but interviews were conducted in “*Igbo*” spoken by respondents in the study areas. The EMIC tool examined the local illness meanings, perceived causes and patterns of distress of the three most common skin NTDs. Six FGDs sessions were held, consisting of one FGD each with 10 adult (>40 years) male and female community participants in Okpoko, one with seven health care workers from the PHCs in the study project sites and another one with eight health workers from two PHCs in two other communities in Ogbaru. Furthermore, one FGD each was held with 8 and 10 patients being treated with NTDs at the two project PHCs. Thus, a total of 52 individuals participated in the FGDs

The sample size for the community survey was calculated using OpenEpi [19]. Assuming that 93%of the community had accurate knowledge of LF [20], a minimum sample of 130 community respondents was adequate at a power of 80% and a precision of 0.05. We interviewed a total of 360 participants (100 and 260 from Ogbakuba and Okpoko communities).

### Instruments and data collection

The survey was carried-out using pilot-tested semi-structured interviewer-administered questionnaires. The community questionnaire had four sections–demographics, knowledge of, attitude to, and care-seeking practices for the NTDs. The questions consisted of factual statements that respondents answered to with ‘yes’, ‘no’ or ‘I don’t know’; or multiple-choice questions with at least one correct answer. The study instruments were reviewed by a group of academics, epidemiologists and public health physicians, social scientists and technical teams from ALM who considered them to have face validity. Pilot-testing of the instruments was performed in one primary health centre and community in Enugu South local government area. The reviews led to modifications in the earlier versions of the instruments.

In each study community, four research assistants who are community health workers with higher education and who had participated in a standardized training session were recruited to administer the questionnaires and to conduct the FGDs and were supervised by research consultants from GLRA. All research assistants completed standardised training sessions on how to administer the questionnaires in both English and Igbo; and those were successful in mock practical sessions were selected to participate in the project. The participants in the FGDs were shown local photographs of typical LF, BU and leprosy lesions as examples of the diseases being discussed. Each disease (i.e., BU, leprosy and LF) and their pictures were shown separately to reinforce the disease being discussed; while the FGD guides based on the EMIC tool was used to ask open-ended questions during each FGD session to identify and inform on the local illness concepts and meanings for the different diseases.

### Data analysis

The questionnaires were entered, cleaned and analysed using SPSS version 20 (Armonk, NY: IBM Corp. USA). Continuous variables were summarized as means ±SD (and where appropriate using median±IQR), and categorical variables as proportions. Categorical variables were compared using the χ^2^ test for proportions. Logistic regression analyses were performed to determine factors associated with knowledge of skin-NTDs. A p-value <0.05 was considered significant. The FGDs were transcribed immediately after the interview, and entered into a word processing package. The transcripts were analysed and using coding and a framework for analysis. The initial meaning units identified were used to construct themes and subthemes and to develop textual descriptions of the experiences. Disagreements in coding interpretation were reviewed and decided by consensus. For each identified theme, data extracts were highlighted on the basis of being representative or interesting illustrations of an emerging issue.

### Ethics statement

The study was approved by the Health Research Ethics Committee of the Anambra State Ministry of Health, Awka, Nigeria. All the participants in the surveys (community participants and health care workers) as well as the FGDs were adults. All participants had written informed consent before inclusion in the study.

## Results

### A. Quantitative Data: Community participants

#### Characteristics of community participants

A total of 353 participants had complete data and were analysed. The mean age of the participants was 38.0 ± 14.1 years and 218 (61.8%) were female. The socio-demographic profiles of the participants are as shown (Table 1).

**Table 1:** Socio-demographic characteristics of the community respondents (N = 353).

Variables	n (%)
Age group (years)	
≤40	217 (61.5)
>40	136 (38.5)
Gender	
Male	135 (38.2)
Female	218 (61.8)
Educational status	
No formal education	24 (6.8)
Primary	93 (26.3)
Secondary	181 (51.3)
Tertiary	55 (15.6)
Marital status	
Single	108 (30.6)
Married	203 (57.5)
Widowed	24 (6.8)
Divorced/Separated	18 (5.1)
Ethnic group	
Igbo	346 (98.0)
Other	7 (2.0)
Religion	
Christianity	333 (94.3)
Islam	3 (0.8)
Traditional religion	17 (4.8)
Occupation	
Employed	88 (24.9)
Unemployed	24 (6.8)
House wife	25 (7.1)
Farmer	49 (13.9)
Unskilled worker	63 (17.8)
Student	42 (11.9)
Other	62 (17.6)
Source of water for drinking	
Tap or Bore hole	300 (85.0)
Rivers or stream	45 (12.7)
Well and others	8 (2.3)
Source of sewage disposal	
Pit-latrine	98 (27.8)
Water closet	227 (64.3)
Open defaecation	25 (7.1)
Others	3 (0.8)
Monthly household income sources	
No defined income	160 (45.3)
Irregular	145 (41.1)
Regular income	48 (13.6)

#### Knowledge of Leprosy, BU and LF

**Fig 1:**
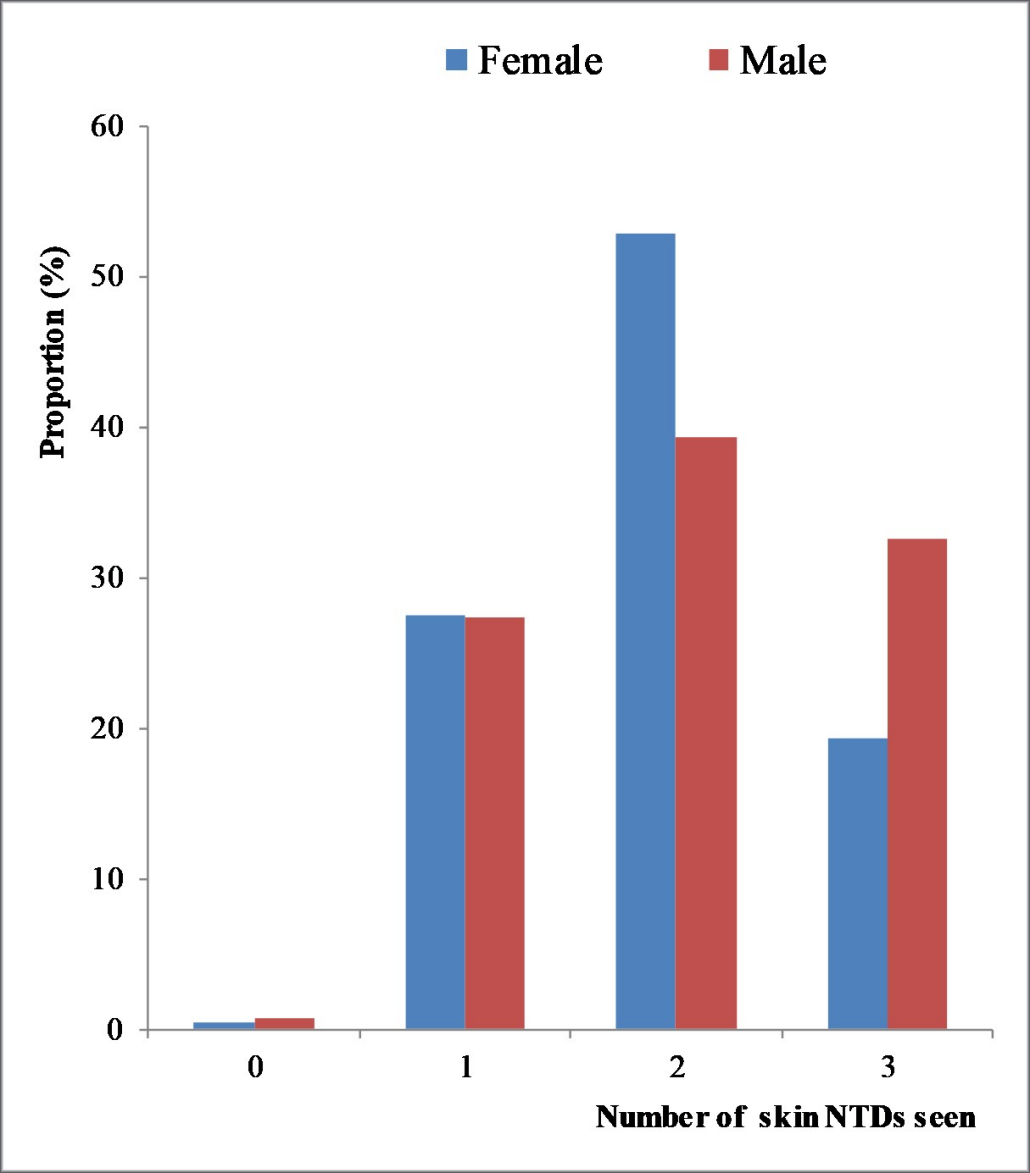
Distribution of participants who had heard or seen individuals with these skin NTDs according to their gender.

The participants were assessed regarding their knowledge of leprosy, BU and LF in their community (Table 2). A total of 351 (99.4%) of the respondents had ever heard or seen a person affected by at least one of these diseases in their community. Furthermore, 236 (66.9%), 324 (91.8%) and 131 (37.1%) of the participants had heard about or encountered individuals with leprosy, BU and LF, respectively. Overall, 2 (0.6%) of the respondents reported not hearing about or seeing someone with any of the skin NTDs, 97 (27.5%) had seen at least one of the three skin NTDs, 168 (47.6%) has heard about or seen at least two of these skin NTDs and 86 (24.4%) had seen or heard about all three skin-NTDs surveyed. Fig 1 shows the distribution of the participants who had heard or seen individuals with these skin NTDs according to their gender. A higher proportion of men compared to women had seen or heard about all three skin NTDs (p = 0.025). The participants first learnt about these skin NTDs mainly from radio jingles 120 (34.0%), followed by family/friends 70 (19.8%).

**Table 2:** Respondents knowledge of skin neglected tropical diseases in Ogabaru, Anambara State, Nigeria.

Variable	Total n (%)	Yes n (%)	No / IDK n (%)
Ever heard of or seen persons with skin neglected tropical diseases in their community	353	351 (99.4)	2 (0.6)
Common skin neglected tropical disease heard or seen by the respondents in their community			
Leprosy	353	236 (66.9)	117 (33.1)
Buruli ulcer	353	324 (91.8)	29 (8.2)
Lymphatic filariasis	353	131 (37.5)	224 (63.5)
Believed that the skin neglected tropical diseases are important health in their community	353	318 (90.1)	35 (9.9)
Participants knowledge of the aetiology of the skin neglected tropical diseases			
Germs	353	167 (47.3)	186 (52.7)
Contact with an affected person	353	74 (21.0)	279 (79.0)
Contact with rivers or swamps	353	48 (13.6)	305 (86.4)
Witchcraft or curse	353	213 (60.3)	140 (39.7)
Poor hygiene	353	149 (42.2)	204 (57.8)
Flies or insect bite	353	61 (17.3)	292 (82.7)
Drinking untreated water	353	81 (22.9)	272 (77.1)
Washing with dirty water	353	64 (18.1)	289 (81.9)
Others	353	5 (1.4)	348 (98.6)
Believed the skin neglected tropical diseases can be transmitted	353	140 (39.7)	213 (60.3)
Believed the skin problems can be sexually transmitted	353	62 (17.6)	291 (82.4)
Believed the skin neglected tropical diseases can be cured	353	336 (95.2)	17 (4.8)
Believed the transmission of the skin neglected tropical diseases can be prevented	353	272 (77.1)	81 (22.9)
Knowledge of how the skin neglected tropical diseases can be prevented			
Covering mouth while coughing or sneezing	353	25 (7.1)	328 (92.9)
Avoiding hand shake with affected persons	353	25 (7.1)	328 (92.9)
Through drinking portable water	353	241 (68.3)	112 (31.7)
Avoiding swimming in rivers or swamps	353	55 (15.6)	298 (84.4)
Sleeping under bed nets	353	70 (19.8)	283 (353)
Sleeping in separate room from affected persons	353	51 (14.4)	302 (85.6)
Avoid sharing of cups with affected persons	353	75 (21.2)	278 (78.8)
Wearing of protective footwear in swampy farm	353	21 (5.9)	332 (94.1)
Others	353	57 (16.1)	296 (83.9)

Also, 318 (90.1%) of the participants believed that these skin NTDs are important health problems in their community. The knowledge of the aetiology of these skin problems varied widely among the participants: only 167 (47.3%) believed they can be caused by germs / infections while others believed they can be caused through contact with affected persons 74 (21.0), contact with rivers and swamps 48 (13.6%) or from insect bite or flies 61 (17.3%). However, 213 (60.3%) believed they can be caused by witchcraft or curse, 149 (42.2%) poor hygiene, 64 (18.1%) drinking untreated water. Furthermore, only 140 (39.7%) believed that any of these skin NTDs could be transmitted, 62 (17.6%) believed it could be sexually transmitted and 336 (95.2%) believed it could be cured. When probed further to describe how the skin NTDs could be cured; 206 (58.4%) indicated this could be achieved through use of modern medicines and wound care, 121 (34.3%) indicated herbal remedies/traditional healers, 12 (3.4%) through prayer/faith healing, and 14 (4.0%) through other means.

Furthermore, 272 (77.1%) of the participants believed that the transmission of these skin NTDs can be prevented. When they were probed further to describe how the diseases can be prevented, 25 (7.1%) reported covering of mouth while coughing/sneezing or avoiding handshake with affected persons, 242 (68.3%) reported drinking of portable water, 55 (15.6%) avoiding swimming in river/swamps, 70 (19.8%) sleeping under bed nets and 51 (14.4%) indicated sleeping in a separate room from affected persons.

#### Community attitudes to leprosy, BU and LF

A total of 315 (89.2%) of the participants agreed or strongly agreed that leprosy, BU and LF are serious skin problem in their community, 187 (53.0%) agreed or strongly agreed that they at risk of acquiring any of these skin NTDs in their community, and 324 (91.8%) agreed or strongly agreed that their community needs to be actively engaged in the control of these skin NTDS (Table 3). The participants’ attitude to being diagnosed with any of these skin NTDs mainly includes; fear 139 (39.4%), sadness or hopelessness 98 (27.8%), shame 68 (19.3%) and surprise 35 (9.9%). Most of the study participants indicated that their main source of advice/help if they develop of any of these skin NTDs will be a healthcare worker 173 (49.0%), others indicated that they would seek help from their spouse 45 (12.7%), parents 43 (12.2%) or a close friend 22 (6.2%).

**Table 3:** Community attitude to neglected tropical diseases of the skin in Ogbaru (N = 353).

Variable	n (%)
Attitude to seriousness of skin neglected tropical disease as an illness in their community	
Strongly agree	223 (63.2)
Agree	92 (26.1)
Neither agree nor disagree	22 (6.2)
Disagree	15 (4.2)
Strongly disagree	1 (0.3)
Respondents attitude of being at risk of acquiring the skin NTDs in their community	
Strongly agree	51 (14.4)
Agree	136 (38.5)
Neither agree nor disagree	59 (16.7)
Disagree	64 (18.1)
Strongly disagree	43 (12.2)
Respondents attitude to active community engagement for skin NTDs control in their community	
Strongly agree	177 (50.1)
Agree	147 (41.6)
Neither agree nor disagree	19 (5.4)
Disagree	9 (2.5)
Strongly disagree	1 (0.3)
Respondent’s reaction to being diagnosed with any of the skin NTDs	
Fear	139 (39.4)
Surprise	35 (9.9)
Shame	68 (19.3)
Sadness or hopelessness	98 (27.8)
Others	13 (3.7)
Respondents’ source of advice/help if s/he develops any of the skin NTDs	
Healthcare worker / doctor	173 (49.0)
Spouse	45 (12.7)
Parent	43 (12.2)
Close friend	22 (6.2)
No one	12 (3.4)
Others	58 (16.4)

#### Community care-seeking practices for leprosy, BU and LF

The participants were asked to indicate the first place they would seek care if they develop any of the skin NTDs: 197 (55.8%) indicated the health centre/hospital, 91 (25.8%) traditional healer/herbalist, 35 (9.9%) indicated pharmacy/patent medicine vendor and 28 (7.9%) indicated prayer houses (Table 4). The participants who indicated that they would not go to a hospital first after developing any of the skin NTDS were asked the reasons: 47 (30.1%) indicated that it is due to high costs of hospital services and 52 (33.3%) believed that she/he will get better care for the problem elsewhere. However, most of the participants 216 (61.2%) indicated that they would seek care in a health facility as soon as they realise the skin problems has started. Overall, 144 (40.8%) of the participants rarely visited or had never visited a health facility for their illness, but most of them 332 (94.1%) were interested in being taught self-care practices for the management of skin NTDs. In addition, at the time of the survey, 215 (60.9%) of the participants reported that they were regularly sleeping under an insecticide treated bed net.

**Table 4:** Community Care-Seeking Practices for Skin Neglected Tropical Diseases in Ogbaru (N = 353).

Variable	n (%)
First place of care-seeking if the respondent develops any of the skin NTDs	
Hospital / health professional	197 (55.8)
Traditional healer / herbalist	91 (25.8)
Pharmacy / Patent Medicine vendors	35 (9.9)
Prayer houses / faith healing	28 (7.9)
Others	2 (0.6)
Why the health facility/Hospital is NOT the preferred first choice of care (N = 156)	
Costs (it is expensive)	47 (30.1)
Difficulties with transportation	2 (1.3)
Do not trust the health care workers	7 (4.5)
Do not like the attitudes of health care workers due to stigmatisation	8 (5.1)
Overlapping work hours with health facility working hours	2 (1.3)
Believe s/he will get better treatment elsewhere	52 (33.3)
Others	38 (24.4)
Point when the respondent will go to a health facility if he/she develops a skin NTD	
When home treatment on her/his own did not work	81 (22.9)
When the problem has lasted for at least one week	9 (2.5)
When the problem has lasted for two to four weeks	20 (5.7)
As soon as s/he realise the skin NTD has started	216 (61.2)
I will NOT go to a health facility to seek care	22 (6.2)
Others	5 (1.4)
How frequently does the respondent visit the health facility (hospital) when ill	
Always	57 (16.1)
Sometimes	152 (43.1)
Rarely	122 (34.6)
Not at all	22 (6.2)
Interest in being taught self-care practices for skin NTDs	
Yes	332 (94.1)
No	21 (5.9)
Currently or regularly sleep under an insecticide-treated bed net	
Yes	215 (60.9)
No	138 (39.1)

#### Factors associated with a belief in herbal cure of the NTDS surveyed

The relationship between the demographic characteristics of the participants, knowledge of the aetiology and a belief in herbal cure of the NTDs surveyed are as shown (Table 5). Gender (p = 0.075) and the mean number of NTDs known or heard of by the respondents (p = 0.744) were not associated with a belief in the efficacy of herbal remedies in the management of the skin NTDs. In univariate analyses, age group (p = 0.002), educational status (p <0.001), belief that the NTDs are caused by germs (p <0.001), belief that the NTDs can be caused by contact with affected persons (p <0.001), belief that the NTDs can be caused by contact with rivers and swamps (p <0.001), belief that the NTDs can be caused by witchcraft / curse (p <0.001), belief that the NTDs can be caused by poor hygiene (p <0.001), belief that the NTDs can be caused by flies / insects (p <0.001), belief that the NTDs can be caused by drinking dirty water (p <0.001), and belief that the NTDs can be caused by washing with dirty water (p = 0.011) were associated with a belief in the efficacy of herbal remedies in the management of the NTDs. However, in multivariable logistic regression analysis, belief that the NTDs are caused by witchcraft/curse (aOR 3.1, 95% CI 1.6–5.8) was an independent predictor of belief in the efficacy of herbal remedies in the management of the NTDs. However, belief that the NTDs are caused by germs (aOR 0.3, 95% CI 0.2–0.6), belief that the NTDs can be caused by contact with affected persons (aOR 0.2, 95% CI 0.08–0.5), belief that the NTDs can be cause by contact with rivers and swamps (aOR 0.3, 95% CI 0.08–0.9) were found to be independently associated with a decreased belief in efficacy of herbal remedies in the management of the NTDs (Table 5).

**Table 5:** Logistic regression analysis of factors associated with a belief in herbal cure for the neglected tropical diseases surveyed.

Variables	Crude OR (95% C I)	Adjusted OR (95% C I)	Adjusted P-value
Older age group (>40 year)	2.0 (1.3–3.1)	1.0 (0.9–1.1)	0.260
Male gender	1.5 (0.9–2.4)	1.4 (0.8–2.5)	0.215
Completed primary education	6.9 (2.2–21.2)	1.3 (0.4–4.0)	0.607
Completed secondary education	6.4 (2.6–15.7)	0.8 (0.3–2.6)	0.761
Completed tertiary education	3.2 (1.3–7.4)	0.9 (0.2–3.7)	0.842
Mean number of NTDs the respondents were aware of or had heard of.	0.9 (0.7–1.3)	0.9 (0.6–1.3)	0.633
Belief that the NTDs are caused by germs / infection	0.1 (0.09–0.2)	0.3 (0.2–0.6)	<0.001
Belief that the NTDs can be caused by contact with affected persons	0.2 (0.7–0.3)	0.2 (0.08–0.5)	0.001
Belief that the NTDs can be caused by contact with rivers and swamps	0.2 (0.05–0.4)	0.3 (0.08–0.9)	0.036
Belief that the NTDs can be caused by witchcraft / curse	5.0 (2.9–8.6)	3.1 (1.6–5.8)	0.001
Belief that the NTDs can be caused by poor hygiene	0.2 (0.1–0.4)	0.6 (0.3–1.3)	0.192
Belief that the NTDs can be caused by flies / insects	0.1 (0.02–0.3)	0.4 (0.09–1.5)	0.154
Belief that the NTDs can be caused by drinking dirty water	0.2 (0.1–0.4)	0.3 (0.09–1.1)	0.068
Belief that the NTDs can be caused by washing with dirty water	0.4 (0.2–0.8)	1.5 (0.4–5.6)	0.508

OR = odds ratio; 95% CI = 95% Confidence Interval

### B. Quantitative Data: Health Workers Survey

A total of 15 female health care workers were interviewed [7 (46.7%) worked in the project and 8 (53.3%) in the control health facility]. Six (40.0%) were nurses, 8 (53.3%) were community health workers and 1 (6.7%) was a community volunteer; all the health workers offer primary care to patients. The mean age of the health care workers was 41±8.1 years, and they have worked for a mean 12.9±8.2 years attending to a mean 7.5±7 patients daily. Overall, 9 (60%) of them were young (<40 years). All the health workers had ever heard of or attended to an individual with at least one of the three NTDs surveyed. Also, 12 (80%), 15 (100%) and 13 (86.7%) of the health care workers had heard about or attended to persons with leprosy, BU and LF, respectively.

Overall, 10 (66.7%) of the health workers considered these skin NTDs as important health problems in their community. Also, 14 (93.3%) of them affirmed that these skin problems are caused by germs; while others believed they can be caused through contact with affected persons 5 (33.3), contact with rivers and swamps 11 (73.3%) or from insect bite or flies 3 (20%). However, 5 (33.3%) believed they can be caused by witchcraft or curse, 12 (80%) poor hygiene, 5 (33.3%) drinking untreated water, and 3 (20%) through the bite of lies or insects. Furthermore, only 9 (60.0%) believed that any of these skin NTDs could be transmitted through contact, 1 (6.7%) believed it could be sexually transmitted and 14 (93.3%) believed it could be cured. When probed further to describe how the skin NTDs could be cured; all 15(100%) indicated this could be achieved through use of modern medicines and wound care, 5 (33.3%) indicated herbal remedies/traditional healers and 1 (6.7%) through prayer/faith healing and 5 (33.3%) through injections. Furthermore, 14 (93.3%) of the health workers believed that the transmission of these skin NTDs can be prevented. When they were probed further to describe how the diseases can be prevented, 3 (20%) reported covering of mouth while coughing/sneezing or 2 (13.3%) avoiding handshake with affected persons, 11 (73.3%) reported drinking of portable water, 8 (53.3%) avoiding swimming in river/swamps, 5 (33.3%) sleeping under bed nets, 8 (53.3%) wearing of protective clothing in swamps, 3 (20.0%) avoid sharing of cups or spoons, 4 (26.7%) sleeping in a separate room from affected persons and 3 (20.0%) indicated immunisation.

#### Health Care Workers Attitudes to Leprosy, BU and LF

A total of 10 (67.7%) of the health care workers agreed or strongly agreed that leprosy, BU and LF are serious skin problem in their community, and 8 (53.0%) agreed or strongly agreed that they are at risk of acquiring any of these skin NTDs from their patients. Furthermore, all 15 (100.0%) the health workers agreed or strongly agreed that patient education and training on self-care practices can contribute to the management of these skin problems, all 15 (100.0%) also agreed or strongly agreed that their community needs to be actively engaged in the control of these skin NTDs and all 15 (100.0%) also agreed or strongly agreed that media campaigns can increase awareness about these skin problems in their community

#### Health Workers Practices in the Care of Leprosy, BU and LF

Overall 13 (86.7%) of the health workers were able to correctly diagnose two of these skin NTDs using photographs of individuals affected by the illness. The health care workers practices in the management of these skin NTDs are as shown (Table 6). Except for checking for sensory loss in an unbroken skin (60.0%), encouraging patients to practice self care (66.7%) and involving family members in caring for the patients (66.7%), most of the health workers (80%) adopted correct practices in the management of the skin NTDS.

**Table 6:** Health care workers practices in the management of skin-neglected tropical diseases in Ogbaru, Anambra State, Nigeria.

Variable	Total n (%)	Yes n (%)	No / IDK n (%)
Total			
Correctly diagnosed at least two of the three skin NTDs	15	13 (86.7)	2 (13.3)
Checks for sensory loss of the area for patient with unbroken skin problem	15	9 (60.0)	6 (40.0)
Does wound clean dressing for patients with open wounds	15	13 (86.7)	2 (13.3)
Correctly combines breathing, elevation and exercise for patients with swellings	15	13 (86.7)	2 (13.3)
Demonstrates limitation of movement by comparing affected and unaffected parts of the body	15	15 (100.0)	0 (0)
Does exercises to improve joint movement in patients with limitation of movement	15	15 (100.0)	0 (0)
Does exercise to strengthen weak muscles in patients with limitation of movement	15	15 (100.0)	0 (0)
Administers assistive technology to improve mobility in patients with limitation of movement	15	15 (100.0)	0 (0)
Provides/ promotes use of protective foot wear in patients with problems of the legs or sole	15	12 (80.0%)	3 (20.0)
Encourages patients to practice self-care	15	10 (66.7)	5 (33.7)
Confirms /encourages patient family members to be involved in their care	15	10 (66.7)	5 (33.3)
Confirms that patient/family are practicing self care	15	8 (53.3)	7 (46.7)

### C. Qualitative Findings

The FGDs found that there is general consistency of the local illness names and meanings of the skin NTDs surveyed.

#### Leprosy: Cause and treatment

Leprosy was commonly named locally either as: “*oya ocha*”, “*oria ocha*” or “*ncha ncha*” which meant the disease that starts as yellowish/whitish/pale/hypopigmented patches on the skin. . . . which if not taken care of can cause damage of the hands and feet. Others averred that leprosy is also locally called “*ekpe nt*a” which means a disease that eats away the hands and feet. All the respondents averred that the disease is very infectious. When they were asked if the disease can be cured, the answer varied with majority of the respondents indicating that it could be only managed via herbal remedies; however female community participants and health workers knew that it could also be treated using modern medicines

“Yes, it can be cured. There are traditional herbal practitioners who know how to treat and cure the disease using herbs and roots. Our major problem in this community is that the skilled native “doctors” who can treat leprosy are all dead, therefore it cannot be treated” (adult male community participants)

“Yes, it could be cured using both herbal remedies and modern medicine. . . . previously such persons were treated with only herbal remedies, but in recent times some of them are treated in leprosy centres in Oji River or Uzuakoli” (adult female community participants)

“No it cannot be cured. . . .. My neighbour had it and visited a traditional centre where herbs were applied but he was never healed”. Some of them were given medical treatment at a leprosy centre but were never cured. A man I know of said he spent over 1 million naira (US$3, 335) to treat the illness but he was not cured, he suffered from the disease until he died in old age” (FGD of LF and BU patients)

“Yes, it is curable with modern medicines…provided they are detected early and started on treatment.. . .Even if such persons are treated and cured, they are not accepted back into the community until he/she bring a written letter from the leprosy centre indicating that he/she is no longer infectious” (FGD community health workers)

#### BU: Cause and treatment

BU was commonly described locally by men, patients and health workers as “*unee ure*” or “*unyi ure*” “a*cha ere*” this means that it is a skin problem that starts as a small thick boil (nodule) and then develops as an ulcer. It is called this name because it is caused by bad/evil people who through charms/ witchcraft/ poison causes affected persons to develop the lesions. Some of the participants indicated that the poison/charm that causes BU is sold for 30 naira ($0.2) in their local markets; and one gets the disease by stepping on it in the farm. Other names used for BU is “*onya nwa dia ala*” which means a persistent ulcer that refuses to heal. However, none of the women surveyed have heard of BU or seen an ulcer lesion suggestive of BU. When photographs of typical cases were shown to them, some of them suggested that they looked like diabetic ulcers. When the FGD participants were asked if the disease can be cured, the answer varied with majority of the respondents indicating that the disease is curable as follows:

“Yes, it can be cured. We have seen persons who were treated and were cured. However, both modern medicines and herbs were used to treat it. Individuals who are skilled in its treatment starts treating it with herbal remedies, then switches to orthodox medicines and when the ulcer granulates, they switch back to herbal remedies until it is cured” (adult male community participants)

“Yes, it is curable? Some persons who get healed from the ulcer re-develop the disease. I first tried local herbal treatment before visiting the health facility, but out of 20 us who were treated by the herbalist, only one of us was healed of his lesion” (FGD of LF and BU patients)

“My skin lesion has been healed since I started the highly effective medicines from your company (organisation). Since I became healed, it does not stop me from doing anything in the community… all I need now is microphone to go and preach to my community that the disease is very curable” (FGD, of LF & BU patients)

“It is curable using orthodox medicines, our patients that have been treated have improved with many healed” (FGD, health care workers)

#### LF (elephantiasis): Cause and treatment

All the FGD groups were able to identify the disease and its local name. This is locally called “*Mgbodo*” or “*Ukwu a ba shoe*” this is because the disease usually causes one of the legs of an affected person to swell such that s/he cannot wear a shoe in one leg. The community and patient FGDs believe that it is caused by evil persons through charms/witchcraft who sends the illness to their enemy. When they were asked if the disease can be cured, the answers varied with majority of the respondent groups indicating that the disease is not curable as follows:

“I have not seen an affected person that was cured… Yes it can be cured. . . . mentions the names three individuals who were treated and were cured. Others concurred that it can be treated with modern medicines and achieve cure” (adult male community participants)

“No, the disease cannot be cured. There is one man in my neighbourhood with this type of swelling, he died with the swelling.. . .It responds to only herbal remedies, but does not heal completely” (adult female community participants)

“I have not seen any person healed from this skin problem” (FGD of LF & BU patients)

“I don’t know how it is treated,. . . . The local treatment for this problem is scarification of the leg with the swelling and application of herbs, if it did not decrease in size, it indicates that this was what was caused by poison or witchcraft by other persons” (FGD, health care workers)

#### Distress faced by individuals with leprosy, BU or LF

In the study communities, affected individuals face a number of distresses including physical, psychological, economic and social distress as a result of their illness. These distresses are worse-off for persons with leprosy who are rejected by their community and family members, because of their illness. Moreover, those with deformities are unable to work/farm, even if they are able to farm, they are unable to sell their produce in the village markets. Also, LF and BU patients have better acceptance in the community and are allowed to participate in community events. Health workers suggested that integrating leprosy, LF and BU care in PHCs may lead to loss of clients particularly women and children who regularly come for maternal and child health services.

“We avoid any form of contact with them in this community–even if he/she (person with leprosy) is your brother or sister. We do not share a plate of food. S/he uses a separate cup, plate and spoon. We don’t share their clothing, if one wears their clothing, he/she will be infected. If a school-age child is affected by leprosy, the community stops him or her from going to school. They are discriminated and stigmatised. . .you cannot share a bench with an affected person when discussing with them” (FGD; community participants)

“For BU and LF, people believe that these diseases are not infectious and they are caused by evil persons. We generally show mercy/pity to them. They are not stigmatised like persons with leprosy. LF and BU prevent women from getting married; the problem does not prevent them from attending community meetings. They are allowed to go to market and they are allowed to participate in business. They participate in village gatherings / village square meetings, however, when children see them, they run away” (FGD; community participants)

“The diseases (Leprosy BU, and LF) does affect children. In fact, if the teacher confirms the diagnosis, the teacher will drive him/her (the child) away from the school” (FGD; community participants and health workers)

“Due to bad smell, all your friends and close associates will stop coming close to you (BU patient).. . . . It prevents me from going to school due to limitations in movement (LF patient). I am 19 years old now, since I started primary One and… I stopped schooling after the ulcer developed. Even though I am healed now and can walk, I am ashamed to return to school” (FGD, BU and LF patient)

“BU and LF patients are not discriminated against, but it affects their daily living (BU patients have bad odour from their ulcer) and LF patients (inability to walk). We will not avoid or drive away an individual/student with a presumptive skin lesion suggestive of leprosy that comes to our health centre, but we will advice that such individuals or student is referred to a centre where they can be treated for the disease” (FGD, health care workers)

“We feel that rather than including NTDs treatment in PHCs, a small clinic should be created in the local government area headquarters where it will be known that individuals with leprosy, LF, BU are treated there and they can/will get better. This is because any PHC that is known to receive and treat persons with leprosy, BU or LF in the community will lose their other clients particularly pregnant women coming for antenatal care and children coming for immunisation” (FGD, health care workers)

## Discussion

There was an average level of awareness of the skin NTDs in the community with a quarter of the participants being aware of all three diseases. Less than half of the participants knew that these lesions were due to germs/infection; more than two-thirds indicated that it can be caused by witchcraft/poison. Only one-fifth knew that sleeping under a bed net may be protective. There was a relatively strong positive attitude towards improving skin NTD control. Although more than half of the respondents would visit a health facility first for a suspicious skin lesion, a quarter of the participants would still consult a herbal practitioner first–and high costs of services and the belief that they would receive better services elsewhere are the main barriers. Although health workers knowledge of aetiology of the skin NTDs was very good, there was still some lack of clarity regarding risk factors of the skin NTDs such as close contact with affected persons, regular contact with streams or swampy areas, belief in poison/witchcraft and drinking untreated water and poor hygiene. Although all the health workers knew that modern medicines and wound care could cure the skin NTDs, a third of them also believed that herbal products are useful; and some believed that immunisation is a preventative measure. Furthermore, most health care workers adopted good practices for the skin-NTDs, but weaknesses in self care practices were noted. The local illness meanings indicate that these diseases derive its name mainly from either the clinical presentation or the wrong beliefs in the aetiology of the skin NTDs. Prominent themes in the FGDs were belief in witchcraft and herbal remedies; as well as the occurrence of physical, social and economic distress. Creating a separate skin NTDs centre was a prominent concern of health care workers.

This study has shown how knowledge of the skin NTDs may influence the demand for such services in an endemic area of Nigeria. It also reveals the contextual circumstances that constrain case detection of leprosy, BU and LF as well as mechanisms that may inform their integrated management. In this study, less than half of the respondents knew that these lesions were due to germs/infection; more than two-thirds of community respondents and a third of health care workers indicated that it can be caused by witchcraft/poison. This wrong knowledge of the causes of these skin NTDs is consistent with previous findings for LF and BU; and may lead affected persons to consult traditional medical practitioners and faith healers for help [16–17,20–22]. Thus, among the respondents, belief in the efficacy of herbal remedies and faith healing remains a consistent theme in the community. The implication of these findings indicate that medical pluralism contributes to delays and interruptions of care along the care cascade for NTDs, and for effective integration of management of leprosy, BU and LF, there is a need for sustained community empowerment through awareness creation using the mass media in other to change their existing beliefs about these diseases and create demand for their services.

Furthermore, less than one-fifth of the participants correctly knew that wearing of protective clothing, sleeping under an insecticide-treated bed net, vaccination and avoiding swimming in rivers and swamps could help in the prevention of the skin NTDs (BU). Some of the respondents inappropriately perceived that wearing a mask to cover their nose and mouth as well as drinking potable water could prevent the skin NTDs. Thus, despite the awareness of skin NTDs in the community, the respondents will undertake inappropriate care-seeking behaviour. Some of the observations in this study differ from those of Akoachere et al., in Cameroun who found that almost half (49.4%) of their respondents thought that BU could be transmitted from one person to another [23], but our finding agrees with those of a report from Benue, Nigeria that showed that one-fifth of LF patients believed the disease could be transmitted through contact [22]. The implication of this finding is that community education on the correct aetiologic causes of these illnesses needs to go hand-in-hand with education on means of preventing these skin NTDs and where to seek care for suspicious lesions. In addition, adequate training and re-training of health care workers on the management or appropriate referral of these NTDs needs to be carried out.

Although the majority of community respondents reported that they would first seek care from a hospital/health professional if they develop a suspicious skin lesion suggestive of any of the skin NTDs, the prominent role of patent medicine vendors, traditional practitioners and faith healers is another important observation of this study. Although in Nigeria, most individuals are not covered by the national health insurance scheme; most individuals have access to a primary health centre where they could seek medical advice. Despite the availability of primary health care centres, the community respondents indicated that they would not go there first due to high costs, belief they would get better services elsewhere, lack of trust in the health system and poor attitude of the staff. Thus, the understanding that financial and structural barriers are also an impediment to early access of skin NTD services in our setting are in keeping with other studies from Nigeria and elsewhere [15–16]. Although the provision of free medication and wound care packs should address most of the direct cost issues, patient and community education will be needed to address beliefs around causation and transmission, strengthen self-care practices and promote the inclusion of family and social support systems.

Furthermore, as has been done by the BU control programme in Nigeria, there is a need to identify and engage informal orthodox and unorthodox health care providers e.g. patent medicine vendors, traditional/herbal healers and prayer houses who can play a key role in case detection of the skin NTDs in endemic communities [24]. This is because these informal providers first see a substantial proportion of these cases, and early case detection will reduce their risk of deformities and disabilities as well the high direct and indirect costs they may incur along the way. In addition, we found that belief in witchcraft/curse as the cause of the skin NTDs was an independent predictor of the belief in efficacy of herbal remedies/traditional healers. Also, belief that the skin NTDs was caused by germs, or from exposure to swamps or contact with affected persons, respectively were inversely associated with the belief in efficacy of herbal remedies/traditional healers. This indicates that having the correct knowledge of the aetiology or risk factors of these skin NTDs lowers the likelihood of community participants believing in the ability of these informal providers and therefore, consulting them when they develop suspicious skin NTDs. This reinforces the need for improved community education prior to the intervention in order to improve their knowledge of the NTDs and where to seek appropriate care in the community. Moreover, as some of these skin NTDs occur in children, there is a need to consider and carry-out appropriate training of health care workers and school teachers on early detection of leprosy, LF and pre-ulcer BU lesions in endemic communities; further studies exploring these possibilities should be undertaken [15].

In this study, we found that a very high proportion of the respondents (94%) indicated that they would be interested in being taught self-care in the management of the skin NTDs. Also, two-thirds of the health care workers indicated that they encouraged their patients to practice self-care; with about half of all health workers confirming that patient/family members of patients with skin NTDs are practicing self care. This high enthusiasm showed by the community participants and health care workers regarding self-care practices for skin NTDs suggests that they would be happy to support such practices in their community. Thus, there is a need to develop a simple culturally-sensitive self-care guide for the management of skin NTDs which should be used both for health workers education and community engagement in endemic settings. This self-care practice guide could be adapted from the standardised guide for health promotion and empowerment of people affected by neglected tropical diseases [25].

The findings of the FGD strengthened our quantitative finding of high beliefs in witchcraft and poison as the cause of the skin NTDs and the belief in efficacy of herbal remedies. In addition, the community and patients had very poor knowledge of individuals with skin-NTDs who were treated and achieved cure. Furthermore, we found that stigma and discrimination was highest for individuals with leprosy and less so for those with BU and LF. As defined by Weiss *et al*. for NTDs, the hidden distress model distinguishes enacted stigma from felt stigma [12, 18]. However, various measurements or definitions of stigma beyond the hidden distress model have been used in NTD-related stigma research, making direct study comparisons difficult [26]. Our study found some gender dimensions in the distress individuals with the skin NTDs may encounter; for example, while men were more concerned about limitations on their economic opportunities, women focused more on the social impact especially the prospects for marriage and family [27]. Previous studies in Ghana and Sri Lanka have shown the impact of stigma on patients with LF and BU, and have also helped to document this aspect of the hidden burden of these diseases [28–29]. Our finding suggests that in integrating management for leprosy, BU and LF; there is a need to address both the shared stigma between these diseases and the specific concerns about leprosy in our setting.

Finally, health workers who participated in the qualitative survey complained that the integrating the management of skin NTDs with PHC services may interfere with the delivery of maternal and child health services because of the unsightly nature of the lesions and the high community stigma about the NTDs. Also, they suggested that a separate NTD clinic needs to be created in affected local government area (for NTDs only). There is a need to further explore this concern raised by the health care workers–particularly how it may interfere with antenatal care attendance by pregnant women and immunisation visits for children. Moreover, broader stakeholder engagement is needed to identify the strategies to integrate the management of morbidity these NTDs in Nigeria and beyond. For example, there is a need to assess the perspectives of current patients affected with varying NTDs on the need to have a separate NTD clinic. Further lessons can be drawn from a recent study on the feasibility of integration of self-care for filarial lymphoedema into existing community leprosy self-help groups in Nepal [30]. Thus, integrated management of skin NTDs provides opportunity not only for morbidity impairments but for integration into existing patient support groups of specific NTDs [30–32]

This study has some strengths and limitations. First, a key strength of this study was that it was carried out in a setting that is co-endemic for the skin-NTDs (leprosy, BU and LF) where a pilot project on integrated morbidity management of these diseases is being carried-out and where little or no literature is available. Second, methods triangulation and the breadth of participants in the FGDs strengthened the validity of our findings because it ensured wider representation of community experiences and views on the skin NTDs thereby increasing its potential for relevance in other settings with a high burden of skin NTDs. Third, this is the first study of which we are aware that has explored community knowledge, attitudes and practices in relation to leprosy, BU and LF in any co-endemic population. Therefore, the findings of this study could serve as a guide to programme managers and health policymakers in designing culturally-sensitive community education and engagement programmes in the promotion of integrated management of these skin-NTDs. However, we refrained from undertaking knowledge scoring of the community respondents and health workers as their responses to the questionnaire were for three different skin NTDs. In addition, we cannot make any causal inferences based on the findings of our quantitative analysis. Moreover, interviewer and response bias in situations whereby the research assistant or participant consciously or subconsciously gave cues or answers influenced by the other cannot be excluded. Our qualitative study validates the findings of this study and improved upon these limitations.

In conclusion, our study helped quantify the information gaps that need to be addressed in order to create demand for integrated skin NTDs services for leprosy, BU and LF in an endemic setting in Nigeria. Individual, structural and socioeconomic challenges to access and delivery of services were identified. Community and health care workers’ empowerment and engagement through outreach and regular training, respectively may alleviate these challenges. Our study findings will be of value to the Nigeria Ministry of Health and also to WHO and other development partners, who are currently developing tools to support integrated management of skin NTDs especially using a community-based approach.

## Supporting information

S1 Text Study instruments. (PDF)

S1 Data Study data–Community KAP. (XLS)

S2 Data Study data–health workers KAP. (XLS)

S1 Fig Pictures used during the study. (PDF)
