# Supplementary material for: Investigating barriers and challenges to the integrated management of neglected tropical skin diseases in an endemic setting in Nigeria
Source: PLoS Negl Trop Dis. 2020 Apr 30;14(4):e0008248. doi: 10.1371/journal.pntd.0008248 (PMC7217480; doi:10.1371/journal.pntd.0008248)
Supplement: S1 Text — (PDF) [file pntd.0008248.s001.pdf]

Community Knowledge, Attitudes, and Practices about Neglected Tropical Diseases inRural Nigeria *(please use language preferred by respondents)*

code of the respondent ____________ Date: ___ / ___ / ___

Location: A.☐ Urban B.☐ Rural

PART ONE: General and socio-demographic characteristics of the respondents

1. **Gender:** **A.** Male B. Female
2. **Age (year):**……………………………
3. **To which ethnic group do you belong?**

   A. Igbo B. Yoruba C. Hausa D. Other__________
4. **What is your religion?**

   A. Christian B. Muslim C. Traditional religion D. Other _________
5. **What is your current marital status?**

   A. Married B. Single C. Divorced D. Widowed
6. **What is the highest level of education you have completed?**

   A. None D. Tertiary education

   B. Primary education E. Other ____________________

   C. Secondary education
7. **What is your current occupation?**

   A. Employed C. Housewife E. Unskilled worker G. Student

   B. Un-employed/Under-employed D. Farmer H. Other ____________
8. **What is your average household income per month?**

   A. No defined income B. Irregular income of ₦…..C. Regular income of ₦…….
9. **What is your source of water for drinking and domestic use?**

   A. Tap / Borehole B. Well. C. River / stream D. Other (specify)…………..
10. **What type of sewage disposal system do you have at home?**

    A. Pit-latrine B. Water closet C. Cesspit D. Open defaecation E. Other specify)…………..

PART TWO: Knowledge and Awareness (please use language preferred by respondents)

1. **Have you ever heard of / or seen individuals with problems on their skin for example, swelling, change in colour, ulcer or other damages on their skin such as the ones we have in these pictures** *(please show pictures containing common NTDs but do not mention the name of any disease)?*

   ☐ Yes ☐ No ☐ I don't know
2. **What are the names of these skin conditions you have heard of / or seen?**(*tick all mentioned)*

   ☐ Leprosy ☐ Buruli ulcer ☐ Lymphatic filariasis ☐ others (Specify)

3. **Where did you first learn about these skin conditions***(tick all that are mentioned.)*
   a. Newspapers and magazines
   b. Radio
   c. TV
   d. Billboards
   e. Brochures, posters and other printed materials
   f. Health workers
   g. Family, friends, neighbours and colleagues
   h. Religious leaders
   i. Teachers
   j. Don't know
   k. Other (please explain):

4. **Do you believe that these skin conditions are important health problems in your community?**

☐ Yes ☐ No ☐ I don't know

5. **What do you think is the cause of these skin problems?** (*pls tick all mentioned)*

A. ☐Germs B.☐Contact with affected persons C. ☐Contact with rivers/swamps

D. ☐ Witchcraft/Curse E. ☐Poor hygiene F. ☐ flies/insect G. ☐ Drinking dirty water

. H. ☐Washing with dirty water I. ☐ Other (specify)…………………..J.☐I don't know

6.Can these skin problems be transmitted by contact with a person with any of these diseases?

☐ Yes ☐ No ☐I don't know

7. Are these skin problems sexually transmitted?

☐ Yes ☐ No ☐ I don't know

8.Can the skin problems be cured? *(Please if No or I don't know, go to No 9)*

☐ Yes ☐ No ☐ I don't know

9.How can these skin problems be cured? *(please circle as many as mentioned)*

A. Modern medicines and wound dressings **B.**Herbal remedies **C.** Prayers **D**. Injections

E. Other ……………………………………. F. I don't know

10. Do you think that the transmission of these skin problems is preventable? *(If YES go to No 10)*

☐ Yes ☐ No ☐ I don't know

11. How can a person prevent getting these skin problems? *(please circle as many as mentioned)*

A. ☐ Covering mouth and nose when coughing or sneezing **B.** ☐ Avoiding hand shake

C. ☐ Through drinking clean/portable water **D.** ☐ Avoiding swimming in rivers

E. ☐ Through sleeping under bed nets **F.** ☐ Using separate room for the patient

G. ☐ Avoiding sharing cups with affected persons **H.** ☐ Wearing of clothing in swampy farm

I. Others (please specify)……………………………… **J.** ☐ I don't know

PART THREE: Assessment of Community Attitudes to NTDs.

1. In your opinion, do you agree /disagree that these skin problems are serious illnesses in your community?

☐Strongly Agree ☐ Agree ☐Neither agree nor disagree ☐ Disagree ☐Strongly Disagree

2. Do you agree /disagree that you may be at risk of getting these skin problems from your community?

☐ Strongly Agree ☐ Agree ☐ Neither agree nor disagree ☐ Disagree ☐ Strongly Disagree

3. Do you think that your community should be actively be engaged to control these skin problems?

☐Strongly Agree ☐ Agree ☐Neither agree nor disagree ☐ Disagree ☐Strongly Disagree

4. What would be your reaction if you were found out that you develop these skin problems?

A. Fear C. Shame E. Other ______

B. Surprise D. Sadness or hopelessness

5. Who would you talk to / seek help from about your illness if you develop these skin problems?

A. Doctor or other health worker C. Parent E. No one

B. Spouse D. Close friend F. Other ____

PART FOUR: Assessment of Community Care-Seeking Practices for NTDs.

1. **If you find-out that you have developed any of these skin problems, where would you FIRST seek care?**(Please choose only one)

   A. Health centre/Hospital B. Traditional healer C. Pharmacy/Chemist D. Prayer houses F. Others (please specify)…………………………………

2. **If answer to 1 is (B to F), Why was a health facility/Hospital NOT your preferred first choice of care?***(please circle ALL that apply)*

   A. Costs (it is expensive)
   B. Difficulties with transportation (because the distance is far)
   C. I do not trust health worker
   D. I do not like the attitude of health workers (stigma)
   E. I cannot leave my work (overlapping work hours with health facility working hours)
   F. I believe I will get better treatment elsewhere
   G. Other (please explain):______________________________

3. **If you see yourself developing any of this skin problems, at what point would you go the health facility***(please circle only one)*

   A. When treatment on my own does not work?
   B. When the problem has lasted for at least one week
   C. When the problem has lasted for two to four weeks
   D. As soon as I realize the skin problem has started
   E. I will not go the health facility to see any health worker at all

F. Other (please explain):______________________________

4. How frequently do you visit the health centre / facility (name) in your area when you are ill?

A. Always B. Sometimes C. Rarely D. Not at all

5. Would you be interested in being taught how to care for yourself if you or your family member develop any of the skin problems?

☐ Yes ☐ No ☐ I don't know

6. Do you currently have and regularly sleep under insecticide-treated bed nets?

☐ Yes ☐ No ☐ I don't know

Thank you very much for participating in our survey!!

Neglected Tropical Diseases: Health workers' Knowledge, Attitudes and Practices in Nigeria

Part A: (Demographics) Please complete the following questions:

1. Age (years)? ……………………….

2. What is your gender? ☐Male ☐Female

3. What is your professional designation (cadre)?

☐ Nurse ☐ medical doctor ☐ Community health officer ☐ lab personnel ☐ Others: …..……………

4. How many years of work experience do you have? _______________ (years)

5. Level of care offered in your health facility? ☐Primary care☐Secondary care ☐ Tertiary care

6. Type of health facility? ☐ Private / mission health facility ☐Public / government health facility

7. Average number of patients attended to daily? ……………………………

Part B: (Knowledge) Please complete the following questions:

1. **Have you everheard of/ or attended to patients who come to your health facility with problems on their skin for example, swelling, change in colour, ulcer or other damages on their skin such as the ones we have in these pictures** *(please show a picture containing common skinNTDs)*

   ☐ Yes ☐ No ☐ I don't know

2. **What are the names of these skin conditions you have heard of / or attended to?***(tick all mentioned)*

   ☐ Leprosy ☐ Buruli ulcer☐ Lymphatic filariasis ☐ Others(Specify)……

3. **Do you believe that these skin conditions are important health problems in your community?**

   ☐ Yes ☐ No ☐ I don't know

4. **What do you think is the cause of these skin problems?** *(pls tick ALL mentioned)*

   A. ☐Germs B.☐Contact with affected persons C. ☐Contact with rivers/swamps

   D.☐Witchcraft/Curse E. ☐Poor hygiene F. ☐ flies/insect G. ☐ Drinking dirty water . H. ☐Washing with dirty water I. ☐ Other (specify)…………………..J.☐I don't know

5.Can these skin problems be transmitted by contact with a person with any of these diseases?

☐ Yes ☐ No ☐I don't know

6.Are these skin problems sexually transmitted?

☐ Yes ☐ No ☐I don't know

7. Can the skin problems be cured? *(Pleaseif No or I don't know, go to No 9)*

☐ Yes ☐ No ☐ I don't know

8. How can these skin problems be cured? *(please circle as many as mentioned)*

A.☐Modern medicines and wound dressings **B.**☐Herbal remedies **C.**☐Prayers

D. ☐Injections

E. Other ……………………………………. F. I don't know

9. Do you think that the transmission of these skin problems is preventable? *(Please if No or I don't know, go to No 11)*

☐ Yes ☐ No ☐ I don't know

10. How can a person prevent getting these skin problems? *(please circle as many as mentioned)*

A. ☐ Covering mouth and nose when coughing or sneezing **B.** ☐ Avoiding hand shake

C. ☐ Through drinking clean/portable water **D.** ☐ Avoiding swimming in rivers

E. ☐ Through sleeping under bed nets **F.** ☐ Using separate room for the patient

G. ☐ Avoiding sharing cups with affected persons **H.** ☐ Wearing of clothing in swampy farm

I. ☐ Vaccination **J.** Others (please specify)………………… **K.** ☐ I don't know

Part C: (Attitudes) Please complete the following questions:

1. In your opinion, do you agree /disagree that these skin conditions areserious illnesses in your community?

☐Strongly Agree ☐Agree ☐Neither agree nor disagree ☐Disagree ☐Strongly Disagree

2. Do you agree /disagree that you may be at risk of getting these skin problems from your patients?

☐Strongly Agree ☐Agree ☐Neither agree nor disagree ☐Disagree ☐Strongly Disagree

3. Do you agree /disagree that patient education and training on self-care practices can contribute to the management of these skin problems?

☐Strongly Agree ☐Agree ☐Neither agree nor disagree ☐Disagree ☐Strongly Disagree

4. Do you think that affected communities in Nigeria should actively be engaged in the control of these skin problems?

☐Strongly Agree ☐Agree ☐Neither agree nor disagree ☐Disagree ☐Strongly Disagree

5. Do you agree /disagree that media campaigns can increase awareness about these skin problems?

☐Strongly Agree ☐Agree ☐Neither agree nor disagree ☐Disagree ☐Strongly Disagree

Part D: (Practices in the management of NTDs) Please tick the appropriate Box:

Now, I would like to ask you some questions regarding your practices in managing persons with these skin problems. I will like you to look at these pictures again and respond to these questions. (*pleasetick only one)*

	Practices	Yes	No	IDK
1	What are the diagnoses of these patients? Correctly diagnosed two NTDs			
2	For the patient with unbroken skin problem, checking for sensory loss of the area is part of its management			
3	For the patient with wounds, clean dressing is an appropriate way to care for it			
4	For patients with swelling, combining breathing, elevation and exercise can contribute to its management			
5	Limitations of movement can be demonstrated by comparing affected and unaffected parts of the body			
6	For patients with limitations of movement, exercise can improve joint movement			
7	For patients with limitations of movement, exercise can strengthen weak muscles			
8	For patients with limitations of movement, assistive technology (like crutches, cane, wheelchair, etc) can help to improve mobility			
9	Use of protective footwear can contribute to the management of patients with problems of the leg / sole.			
10	Do you encourage your patients to practice self care?			

11. From your experience, are patients family members involved in providing care to patients?

☐ Yes ☐ No ☐ I don't know

12. Are some of your patients with these skin conditions practicing self care? *(Please if Yes, go to 13)*

☐ Yes ☐ No ☐ I don't know

13. What percentage of your patients practice self-care for their skin problem? ………………….(%)

Thank you very much for participating in our survey!!

Assessment of Local illness Meanings and Patterns of Distress in Patients with NTDs in Rural Nigeria

FOCUS GROUP DISCUSSIONGUIDE (COMMUNITY/HEALTH WORKERS)

Hello. My name is ________________ and I am working for The German Leprosy/TB Relief Association. We are conducting this group interview to help us understand from your perspectives what these skin problems (show a picture) mean in your context and the challenges persons who have these problems face in your community. Please feel free to discuss. The information that you provide in this interview although recorded is confidential and will be used only for research. The Discussion will take only 25–30 minutes.

Before we start, let us introduce ourselves:.

For each person:

Gender: A. Male B. Female

Age (years): …………………………….

Occupation……………………………..

I want to us to discuss what the meaning of these illnesses are in the context of your community

a. Leprosy
   - What is the local name of this condition?
   - Why is it called this name?
   - Is it curable? If Yes why? If No..why?
   - Locally, how is it treated?

b. Buruli ulcer
   - What is the local name of this condition?
   - Why is it called this name?
   - Is it curable? If Yes why? If No..why?
   - Locally, how is it treated?

c. Lymphatic filariasis
   - Why is it called this name?
   - Is it curable? If Yes why? If No..why?
   - Locally, how is it treated?

d. Other skin problems?
   - What is the local name of this condition?
   - Why is it called this name?
   - Is it curable? If Yes why? If No..why?
   - Locally, how is it treated?

Also, persons who have this skin problem(s) face or may face various challenges either from the illness itself or how they/families are looked at in the community. I will want us to discuss each of these challenges according to each skin disease.

1. **Let us start with individuals with Leprosy**

What are the problems or features that disturb the well-being of individuals with this disease?

(A list of problems is provided here? (If the group did not mention a category, please prompt)

- **Physical problems**: Pain, Loss of sensation, muscle weakness, Inability to walk
- **Social Problems**: Rejection from family and/or peers, Disrupted education, Inability to get married, Divorce, Non-disclosure, non-participation in community activities
- **Psychological / emotional**: Anxiety, Depression, Fear of Surgery, Embarrassed
- **Economic problems**: (Loss of business/income, Inability to work, inability to access loans, Sale of assets, e.t.c)
- **Others:**

2. OK. What of individuals with Buruli ulcer

What are the problems or features that disturb the well-being of individuals with this disease?

(A list of problems is provided here? (If the patients did not mention a category, please prompt)

- **Physical problems**: wound, swelling, limitation of movement at joints, fever, pain,
- **Social Problems**: Rejection from family and/or peers, disrupted education, Inability to get married, Divorce, Non-disclosure, non-participation in community activities
- **Psychological / emotional**: Anxiety, Depression, Fear of Surgery, Embarrassed
- **Economic problems**: (Loss of business/income, Inability to work, inability to access loans, Sale of assets, e.t.c)
- **Others:**

3. OK. What of individuals with Lymphatic filariasis

What are the problems or features that disturb the well-being of individuals with this disease?

(A list of problems is provided here? (If the patients did not mention a category, please prompt)

- **Physical problems**: Swelling of the affected limbs/scrotum, wound, Fever, Pain,difficulty in walking
- **Social Problems**: Rejection from family and/or peers, Disrupted education, Inability to get married, Divorce, Non-disclosure, non-participation in community activities
- **Psychological / emotional**: Anxiety, Depression, Fear of Surgery, Embarrassed
- **Economic problems**: (Loss of business/income, Inability to work, inability to access loans, Sale of assets, e.t.c)
- **Others:**

4. OK. What of individuals with other skin diseases

What are the problems or features that disturb the well-being of individuals with this disease?

(A list of problems is provided here? (If the patients did not mention a category, please prompt)

- **Physical problems**: Fever, Pain, Loss of sensation, Weakness, Inability to walk
- **Social Problems**: Rejection from family and/or peers, Disrupted education, Inability to get married, Divorce, Non-disclosure, non-participation in community activities
- **Psychological / emotional**: Anxiety, Depression, Fear of Surgery, Embarrassed
- **Economic problems**: (Loss of business/income, Inability to work, inability to access loans, Sale of assets, e.t.c)
- **Others:**

Thanks for your time

Assessment of Local illness Meanings and Patterns of Distress in Patients with NTDs in Rural Nigeria

FOCUS GROUP DISCUSSION GUIDE (PATIENTS)

Hello. My name is _______________ and I am working for The German Leprosy/TB Relief Association. We are conducting this group interview to help us understand from your perspectives what the skin problems mean in your context and the challenges you might have encountered since the problem started. Please feel free to discuss. The information that you provide in this interview although recorded is confidential and will be used only for research. The Discussion will take only 25–30 minutes.

Before we start, let us introduce ourselves:

For each person:

Diagnosis of the patient…………………………..
Gender: A. Male B. Female
Age (year):)? …………………………….
Occupation…………………

I want us to discuss what the meaning of these illnesses are in the context of your community

a. Leprosy
    - What is the local name of this condition?
    - Why is it called this name?
    - Locally, how is it treated?
    - Is it curable? If Yes why? If No..why?
b. Buruli ulcer
    - What is the local name of this condition?
    - Why is it called this name?
    - Locally, how is it treated?
    - Is it curable? If Yes why? If No..why?
c. Lymphatic filariasis
    - What is the local name of this condition?
    - Why is it called this name?
    - Locally, how is it treated?
    - Is it curable? If Yes why? If No..why?
d. Other skin problems (e.g. Yaws)
    - What is the local name of this condition?
    - Why is it called this name?
    - Locally, how is it treated?
    - Is it curable? If Yes why? If No..why?

Also, persons who have this skin problem(s) face or may face various challenges either from the illness itself or how they/families are looked at in the community. I will want us to discuss each of these challenges according to each skin disease.

1. **Let us start with individuals with Leprosy**

What are the problems or features that disturb the well-being of individuals with this disease?

(A list of problems is provided here? (If the group did not mention a category, please prompt)

- **Physical problems**: Pain, Loss of sensation, muscle weakness, Inability to walk
- **Social Problems**: Rejection from family and/or peers, Disrupted education, Inability to get married, Divorce, Non-disclosure, non-participation in community activities
- **Psychological / emotional**: Anxiety, Depression, Fear of Surgery, Embarrassed
- **Economic problems**: (Loss of business/income, Inability to work, inability to access loans, Sale of assets, e.t.c)
- **Others:**

2. OK. What of individuals with Buruli ulcer

What are the problems or features that disturb the well-being of individuals with this disease?

(A list of problems is provided here? (If the patients did not mention a category, please prompt)

- **Physical problems**: wound, swelling, limitation of movement at joints, fever, pain,
- **Social Problems**: Rejection from family and/or peers, disrupted education, Inability to get married, Divorce, Non-disclosure, non-participation in community activities
- **Psychological / emotional**: Anxiety, Depression, Fear of Surgery, Embarrassed
- **Economic problems**: (Loss of business/income, Inability to work, inability to access loans, Sale of assets, e.t.c)
- **Others:**

3. OK. What of individuals with Lymphatic filariasis

What are the problems or features that disturb the well-being of individuals with this disease?

(A list of problems is provided here? (If the patients did not mention a category, please prompt)

- **Physical problems**: Swelling of the affected limbs/scrotum, wound, Fever, Pain,difficulty in walking
- **Social Problems**: Rejection from family and/or peers, Disrupted education, Inability to get married, Divorce, Non-disclosure, non-participation in community activities
- **Psychological / emotional**: Anxiety, Depression, Fear of Surgery, Embarrassed
- **Economic problems**: (Loss of business/income, Inability to work, inability to access loans, Sale of assets, e.t.c)
- **Others:**

4. OK. What of individuals with other skin diseases (………………indicate)

What are the problems or features that disturb the well-being of individuals with this disease?

(A list of problems is provided here? (If the patients did not mention a category, please prompt)

- **Physical problems**: Fever, Pain, Loss of sensation, Weakness, Inability to walk
- **Social Problems**: Rejection from family and/or peers, Disrupted education, Inability to get married, Divorce, Non-disclosure, non-participation in community activities
- **Psychological / emotional**: Anxiety, Depression, Fear of Surgery, Embarrassed
- **Economic problems**: (Loss of business/income, Inability to work, inability to access loans, Sale of assets, e.t.c)
- **Others:**

Thanks for your time
