# Supplementary figures and images for: Investigating barriers and challenges to the integrated management of neglected tropical skin diseases in an endemic setting in Nigeria
Source: PLoS Negl Trop Dis. 2020 Apr 30;14(4):e0008248. doi: 10.1371/journal.pntd.0008248 (PMC7217480; doi:10.1371/journal.pntd.0008248)

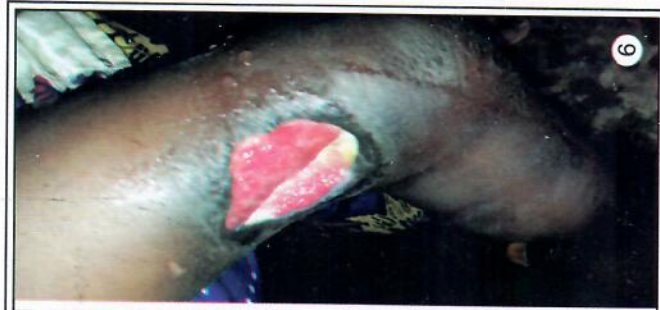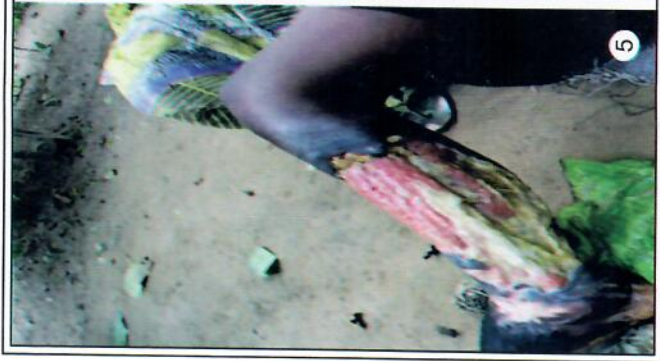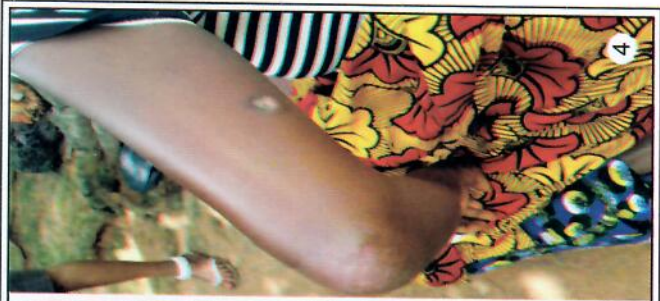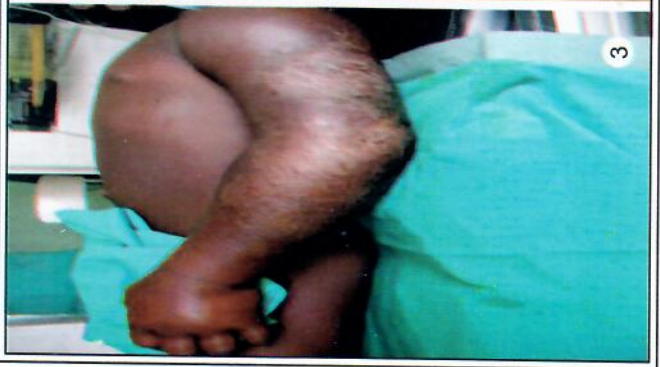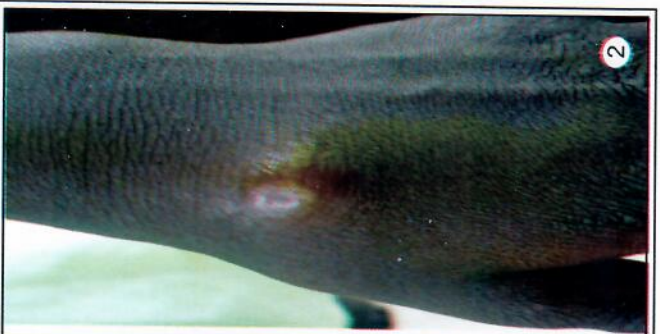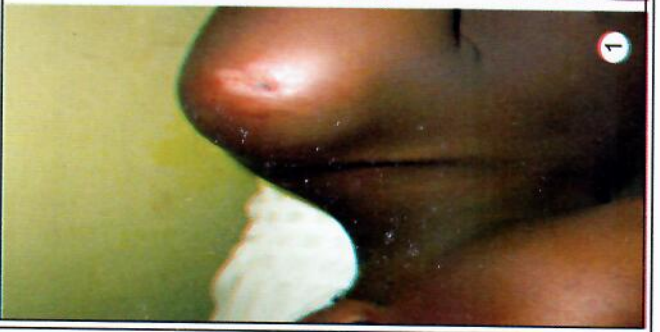

# Some Neglected Tropical Diseases of the Skin in Nigeria

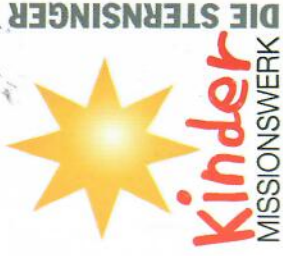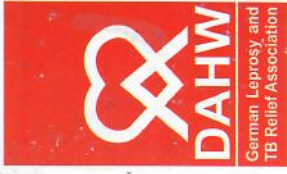

**DAHW Nigeria, 2019**  
07067490052, 08036256424  
(Call Time: 8am – 4pm)

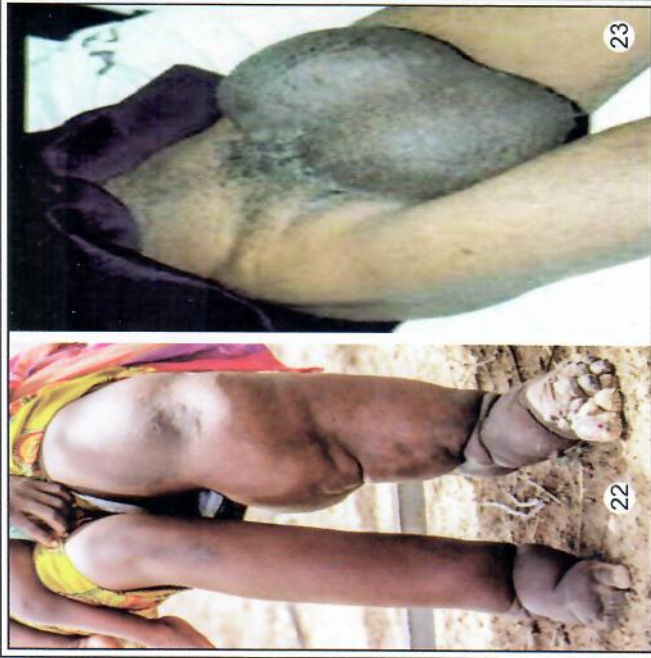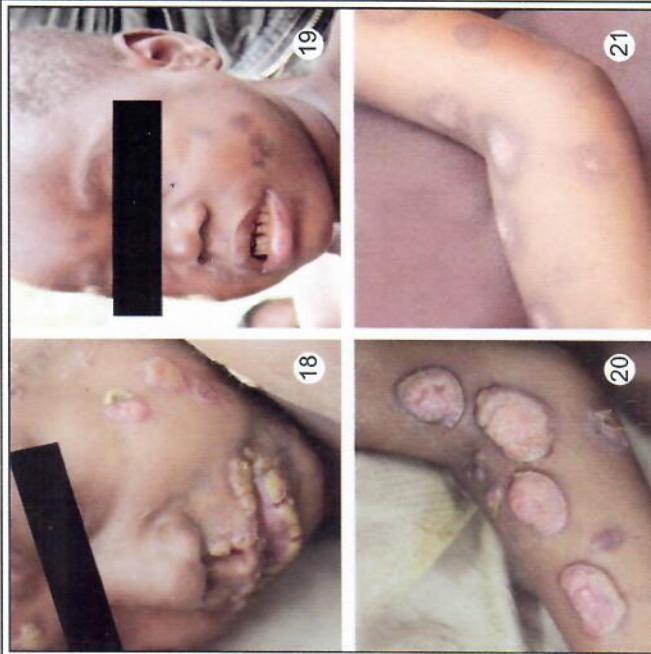

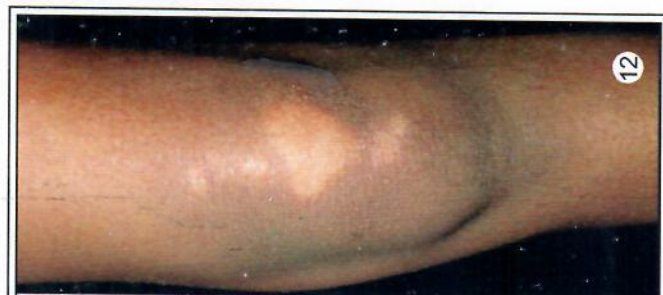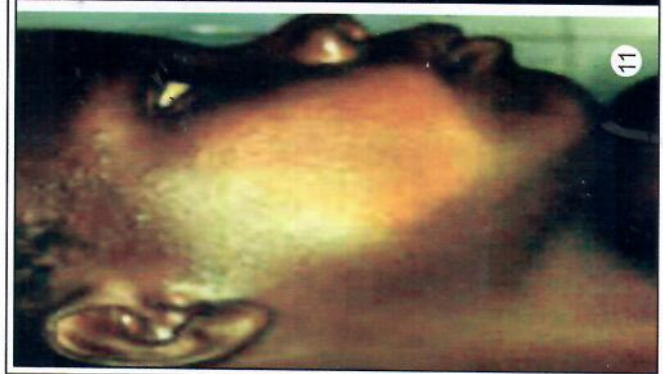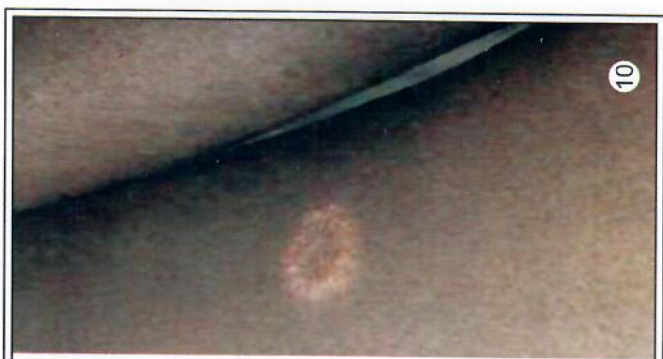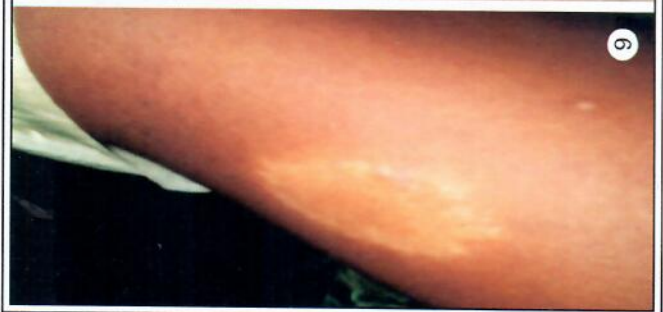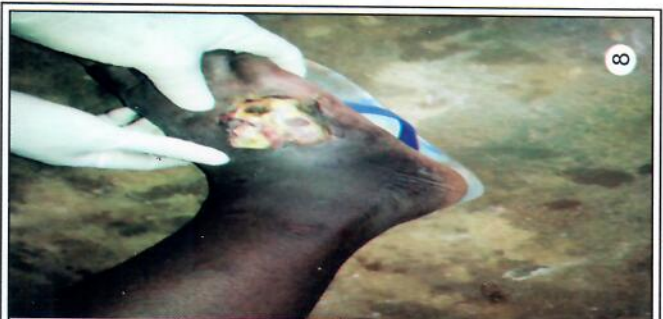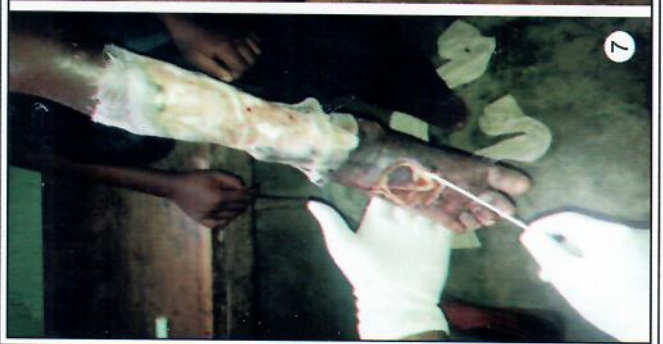

Supplement: S1 Fig — (PDF) [file pntd.0008248.s004.pdf]
